# Frailty and risk of complications in head and neck oncologic surgery. Systematic review and dose-response meta-analysis

**DOI:** 10.4317/medoral.24588

**Published:** 2021-08-19

**Authors:** Mário Luis Tavares Mendes, Claudiane Mahl, Aline Carla Araújo Carvalho, Victor Santana Santos, Diego Moura Tanajura, Paulo Ricardo Martins-Filho

**Affiliations:** 1 Investigative Pathology Laboratory, Federal University of Sergipe, Aracaju, Sergipe, Brazil; 2 Health Sciences Graduate Program, Federal University of Sergipe, Aracaju, Sergipe, Brazil; 3 State University of Health Sciences of Alagoas, Maceió, Alagoas, Brazil; 4 Cesmac University Center, Maceió, Alagoas, Brazil; 5 Centre for Epidemiology and Public Health, Federal University of Alagoas, Arapiraca, Alagoas, Brazil

## Abstract

**Background:**

There is emerging evidence that frail individuals present a decreased physiological reserve, decreased ability to maintain homeostasis, and increased vulnerability to stressors. The concept of frailty has become increasingly recognized as a valuable measure in oncological surgical patients, including those with head and neck cancer. Preoperative screening for frailty may provide an individualized risk assessment that can be used by an interdisciplinary team for preoperative counseling and to improve outcomes. The aim of this meta-analysis was to evaluate the relationship between frailty and the risk of major postoperative complications in frail individuals submitted to head and neck oncologic surgery.

**Material and Methods:**

PubMed, SCOPUS, Web of Science, Google Scholar and OpenThesis were systematically searched to identify studies that evaluated the risk of major postoperative complications in frail individuals undergoing head and neck oncologic surgery. The search was performed on August 31, 2020, without language or date restrictions. Two independent investigators screened the searched studies based on each paper’s title and abstract. Relevant studies were read in full and selected according to the eligibility criteria. Frailty was assessed by modified Frailty Index (mFI-11) and major postoperative complications were measured by the Clavien-Dindo classification. We performed a categorical and dose-response meta-analysis using a random-effects model to evaluate the association between frailty and the risk of major postoperative complications in patients submitted to head and neck oncologic surgery. The results of the meta-analysis were expressed as relative risk (RR) and 95% confidence interval (95% CI). The risk of bias was assessed using the Newcastle-Ottawa Scale (NOS).

**Results:**

Four studies (9,947 patients) were included in this systematic review and meta-analysis. Frail patients presented an increased risk of life-threatening complications requiring intensive care unit (ICU) admission (RR = 4.67; 95% CI 1.54–14.10) and 30-day mortality (RR = 8.10; 95% CI 2.30–28.57) compared to non-frail patients. We found evidence of dose-response trend between mFI-11 and major postoperative complications.

**Conclusions:**

Higher frailty scores are associated with a significant increase in ICU-level complications and 30-day mortality after head and neck oncologic surgery.

** Key words:**Frailty, head and neck neoplasms, postoperative complications, mortality.

## Introduction

Frailty has been characterized as a multidimensional syndrome resulting from cumulative cellular damage over the life course and is identified as a major predictor of adverse outcomes in older subjects (1). Frail individuals present a decreased physiological reserve, decreased ability to maintain homeostasis, and increased vulnerability to stressors. There is emerging evidence that frailty is associated with mortality, falls, worsening disability, hospitalization, and care home admission in cohorts of elderly people (2). In addition, the concept of frailty has become increasingly recognized as a valuable measure in oncological surgical patients, including those with head and neck cancer (3).

The heterogeneity of patients with cancer and the multidimensional effects of both malignancy and surgery underscore the importance of incorporating more comprehensive preoperative assessments in oncology surgery (4). Preoperative screening for frailty may provide an individualized risk assessment that can be used by an interdisciplinary team for preoperative counseling and to improve outcomes. Moreover, frailty stratification can help to plan interventions and to predict a patient’s risk of death or need for institutional care (5).

Several validated diagnostic tools are available to classify and measure frailty. The most common frailty measurement is the phenotypic model proposed by Fried *et al*., which evaluates frailty through five criteria: unintentional weight loss, subjective exhaustion, low grip strength, reduced walking speed, and low levels of physical activity (6). The other widely used tool is the frailty index (FI) proposed by Rockwood *et al*., where frailty is measured by a checklist of clinical conditions and is taken as a consequence of accumulation of deficits (7). The FI was first introduced by the Canadian Study of Health and Aging (CSHA) to provide a standardized definition of frailty using measurable parameters easily ascertained in a clinical setting.

Because the accumulating deficits model is mostly based on the patient’s history, the FI may be a more practical method of clinically assessing preoperative frailty (8). Based on the FI, the modified Frailty Index (mFI) using 11 clinical variables (mFI-11) was created by Saxton and Velanovich which has been proposed for risk stratification, preoperative optimization, and perioperative counseling (9). The mFI-11 evaluates variables related to the history of diabetes mellitus, congestive heart failure, hypertension requiring medications, myocardial infarction, percutaneous coronary intervention and/or stenting or angina, transient ischemic attack, cerebrovascular accident with neurologic deficit, impaired sensorium, chronic obstructive pulmonary disease or pneumonia, peripheral vascular disease or rest pain, and functional status index. The mFI-11 ranges from 0 to 1 and is calculated by dividing the number of factors present for a patient by the number of available factors (n/11). A higher mFI-11 score is representative of more significant frailty (10).

In head and neck surgeries, adverse events are commonly evaluated using the Clavien-Dindo (CD) system, which includes five categories (I-V) of increasing severity based on the clinical treatment of complications. CD grade IV (CD-IV) and CD grade V (CD-V) are considered major postoperative complications and include any life-threatening complication requiring intensive care unit (ICU) admission and death, respectively (11). The knowledge of preoperative risk is important in the reduction of post-surgical complications and costs in health services. We hypothesized that additional accumulated deficits could increase the risk of post-surgical complications in head and neck oncologic surgery. The aim of this dose-response meta-analysis was to evaluate the relationship between frailty and the risk of ICU-level complications and death in frail individuals submitted to head and neck oncologic surgery.

## Material and Methods

This study was conducted following the Meta-Analysis of Observational Studies in Epidemiology (MOOSE) statement (12). Institutional review board approval and informed consent were not required for this systematic review and meta-analysis.

- Research question and eligibility criteria

The present study focused on the following question: Is there a dose-response relationship between frailty and the risk of postoperative complications in head and neck oncologic surgery? Studies were considered eligible if they satisfied the following criteria: (i) they were retrospective or prospective studies; (ii) patients submitted to head and neck oncologic surgery, including upper aerodigestive tract (UADT) (oral cavity, lip, salivary glands, pharynx, larynx, nasal cavity, paranasal sinuses, esophagus), thyroid, parathyroid, associated lymph nodes, soft tissues, and bone (13,14); (iii) major postoperative complications measured by the CD classification; (iv) frailty assessed by mFI-11. We excluded case reports, case series, conference proceedings, scientific meeting abstracts, editorials, and letters to the editor that did not provide original data. Patients submitted to reconstructions of head and neck defects were not included to limit the effect of additional surgical sites and variation in post-surgical care, which may lead to bias in meta-analysis results.

- Search strategy

A systematic search using the PubMed, SCOPUS, and Web of Science databases was performed to identify studies that evaluated the risk of postoperative complications in frail individuals undergoing head and neck oncologic surgery. A grey literature search was conducted using Google Scholar and OpenThesis. The search was performed on August 31, 2020 without language or date restrictions. Studies published in non-English language were translated using professional translation services if necessary. The reference lists of all eligible studies were manually checked to identify additional studies for inclusion. For studies where data were not explicitly reported, the corresponding authors were contacted and asked to provide information. The full electronic search strategy is illustrated in Supplement 1.

- Study selection

Two independent investigators (A.C.A.C. and M.L.T.M.) screened the searched studies based on each paper’s title and abstract. Relevant studies were read in full and selected according to the eligibility criteria. Disagreements between the two reviewers were resolved by consensus or by a third reviewer (P.R.S.M.-F.).

- Frailty and major postoperative complications assessment

Frailty was assessed using the mFI-11, which includes 10 items related to comorbid conditions and one item related to the patient’s functional status (basic and instrumental activities of daily living). To calculate the mFI-11, the presence of each variable equals one point, and the total points for each patient is divided by 11 to obtain the patient’s mFI score (range, 0-1). Patients were classified as non-frail (mFI = 0), pre-frail (mFI = 0.09-0.18), and frail (mFI ≥ 0.27) (15).

Major postoperative complications included CD-IV (ICU-level complications within 30 days after surgery, including unplanned intubations, pulmonary embolism, failure to wean off the ventilator more than 48 hours after surgery, acute renal failure, cardiac arrest requiring initiation of cardiopulmonary resuscitation, acute myocardial infarction, and severe sepsis or septic shock) and CD-V (death).

- Data extraction

Two independent investigators (A.C.A.C. and M.L.T.M.) extracted data from the published reports using a predefined protocol. Information about the study design, eligible population, age and gender distribution, inclusion and exclusion criteria, frailty assessment, and postoperative complications were checked. To evaluate the relationship between mFI-11 and postoperative complications, we extracted from primary studies the number of CD-IV and CD-V complications for each cutoff value for frailty from the mFI-11.

- Risk of bias assessment

The quality of the studies was assessed by two independent reviewers (A.C.A.C. and M.L.T.M.) using the Newcastle-Ottawa Scale (NOS), and included evaluations of the representativeness of the sample size, selection of a comparison group (whether participants were drawn from the same source, e.g., same institution or database), ascertainment of exposure by secure records, outcome of interest not present at the start of the study, outcomes adjusted for age and other possible confounding variables, assessment of postoperative complications by independent blind assessment or record linkage, adequate follow-up period for outcome of interest (30 days), and loss to follow-up unlikely to introduce bias. These items were not merged into a quality score. Instead, the relevant information for each domain was tabulated to allow for greater transparency.

- Data analysis

We performed a categorical and dose-response meta-analysis using a random-effects model to evaluate the association between frailty and the risk of major postoperative complications in patients submitted to head and neck oncologic surgery. The categorical meta-analysis was conducted by pooling the relative risk (RR) for frail patients (mFI ≥ 0.27) compared to non-frail individuals (mFI = 0). Forest plots were used to graphically present the pooled RR and the 95% confidence intervals (CI). P-values lower than 0.05 were statistically significant. Heterogeneity was investigated using the Cochran Q test with a cutoff of 10% for significance and quantified using the I2 index (16). Publication bias was not assessed by inspecting the funnel plot because of the small number of studies included in this systematic review. Leave-one-out sensitivity analysis was performed to assess the robustness of the pooled results. The categorical meta-analysis was performed using Review Manager version 5.3 software (Cochrane Collaboration, Copenhagen).

The multivariate dose-response meta-analysis evaluated the relationship between mFI-11 scores and the risk for major postoperative complications. This analysis was performed using the dosresmeta R package and modelled with both a linear and quadratic curve (17). We used the Hamling method (18) to approximate the covariances by defining a Table of effective counts corresponding to the multivariable adjusted log RR. Dose-specific RR were reported with the corresponding 95% CI.

We used the Akaike Information Criterion (AIC) to compare the fittings of dose−response curves with linear and quadratic models. The AIC value represents how well a model fits the data set, where a relatively low AIC means a better fit than a higher AIC.

## Results

- Study selection

**Figure 1:**
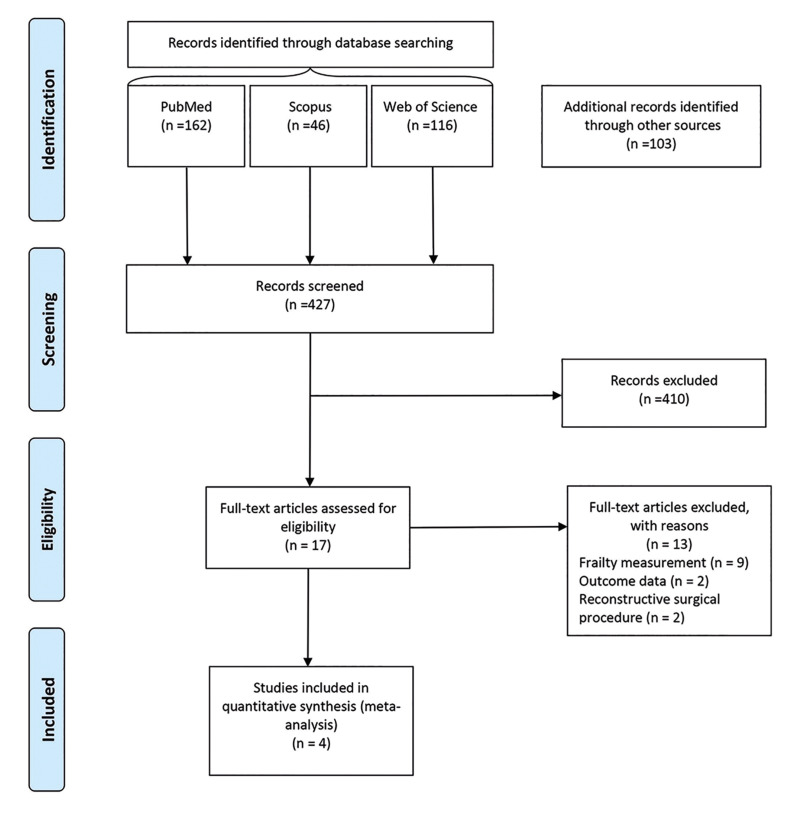
Diagram of the study selection process for the systematic review and meta-analysis.

The initial search located 427 records through electronic databases, of which 162 were collected from PubMed, 46 from SCOPUS, 116 from Web of Science, 100 from Google Scholar and three via a hand-searching. No studies were found in the OpenThesis database. Seventeen studies were potentially relevant and were analyzed in full. After reading the full-texts, 13 studies were excluded due to the frailty or postoperative complications measurements, outcome data, or reconstructive surgical procedure. Finally, four studies satisfied the eligibility criteria and were included in the present systematic review (19,20-22). A flowchart depicting the selection process at each stage is provided in Fig. 1.

- Study characteristics and risk of bias assessment

The studies included in this systematic review reported data from 10,358 patients submitted to head and neck oncologic surgery. However, due to the potential overlapping population, data from 394 patients undergoing total laryngectomy and 17 patients submitted to esophagectomy were excluded. Finally, data from 9,947 patients were analyzed in this study.

All studies had a retrospective design and included data from the American College of Surgeons National Surgical Quality Improvement Program (ACS-NSQIP). The ACS-NSQIP is a national risk- and case-mix-adjusted database that collects deidentified information to measure and improve the quality of surgical care. This database includes data on preoperative risk factors, intraoperative variables, and 30-day postoperative mortality and morbidity outcomes for patients undergoing major surgical procedures in both the inpatient and outpatient setting.

The mean age of the patients ranged from 55 to 63 years and most of them were male. Most patients were submitted to surgery for UADT neoplasms. Only the study performed by Adams *et al*.(19) included patients undergoing thyroidectomy, tonsillectomy and parotidectomy. The primary characteristics of the studies are listed in Table 1. All studies included in this systematic review had a low risk of bias (Supplement 2).

- Risk of postoperative complications

**Figure 2:**
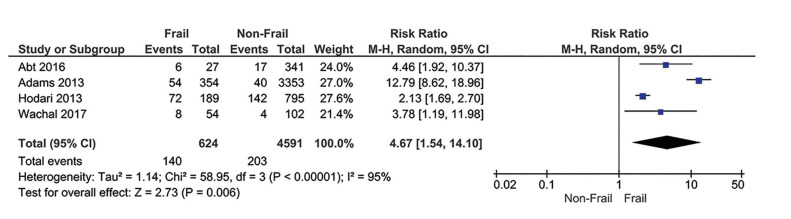
Categorical meta-analysis showing the risk for CD-IV complications in frail individuals.

All studies included in this meta-analysis provided data on CD-IV complications. One hundred and forty (22.5%) frail patients (mFI-11 ≥ 0.27) and 203 (4.4%) non-frail patients (mFI-11 = 0) had CD-IV complications. Frail patients presented an increased risk of CD-IV complications compared to non-frail patients (RR = 4.67; 95% CI 1.54-14.10; I2 = 95%) (Fig. 2).

**Figure 3:**
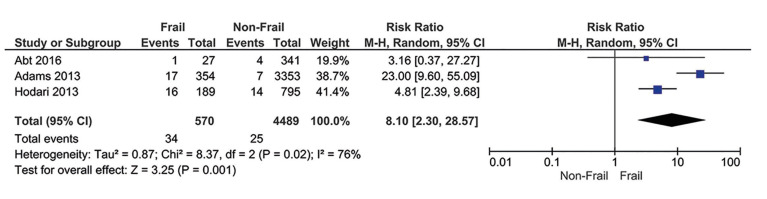
Categorical meta-analysis showing the risk for death after surgery in frail subjects.

Three studies (19,20,21) reported data on 30-day mortality for patients submitted to head and neck oncologic surgery. There were 34 (6%) deaths among frail patients (mFI-11 ≥ 0.27) and 25 (0.6%) deaths among non-frail patients (mFI-11 = 0). Frail patients presented an increased risk of death compared to non-frail patients (RR = 8.10; 95% CI 2.30-28.57; I2 = 76%) (Fig. 3).

- Dose-response meta-analysis

We found evidence of a linear and quadratic dose-response trend between mFI-11 and major postoperative complications (Supplement 3, Supplement 4). We tested the best-fitting model using the AIC, and the linear function model was more appropriate to predict the CD-IV complications and 30-day mortality. Dose-specific RR with the corresponding 95% CI are reported in Table 2.

**Table 1:**
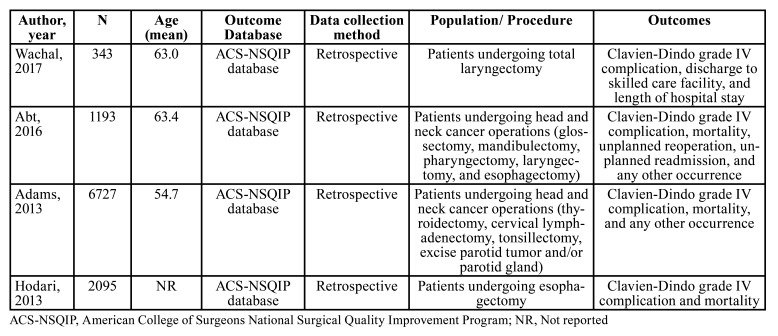
Characteristics of included studies evaluating the association between frailty and major complications after head and neck oncologic surgery.

**Table 2:**
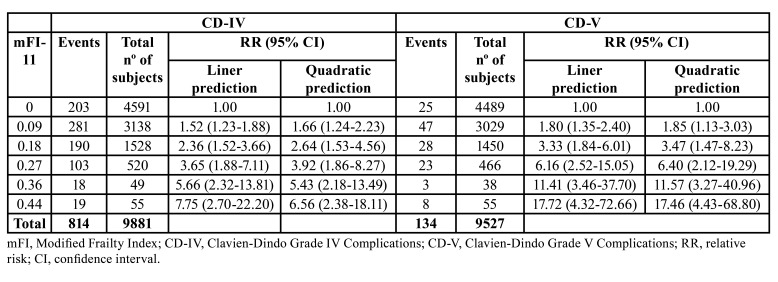
Dose-response meta-analysis between mFI-11 and relative risk for major postoperative complications after head and neck oncologic surgery.

## Discussion

Head and neck cancer accounts for more than 900,000 cases and 370,000 deaths worldwide annually (23). Successful outcomes depend upon appropriate surgical management, treatment of concurrent illnesses, and minimization of postoperative complications. The identification of frail patients with a high risk of postoperative morbidity and mortality is important for adequate clinical and surgical decision making. Measurement tools for preoperative frailty assessment show a promising ability to predict perioperative morbidity, mortality, and to guide patient selection and intervention. The mFI-11 has been suggested as a predictive tool to identify individuals at risk of postoperative complications based on preoperative comorbidities. Although studies have shown that mFI-11 is more strongly associated with morbidity and mortality than surgical wound class, ASA, age, and other frailty assessments (19,24), the value of frailty as a predictor of adverse events has remained little explored in head and neck oncologic surgery. This systematic review and dose-response meta-analysis analyzed the current evidence on the risk of postoperative complications in frail individuals submitted to head and neck oncologic surgery.

Our meta-analysis showed an increased risk of CD-IV complications and 30-day mortality in frail patients after head and neck oncologic surgery. The ability to predict relevant postoperative outcomes is one of the most important characteristics of any risk stratification variable or system. Risk prediction plays an important role in recognizing patients at high risk of complications and in implementing appropriate treatments, thereby preventing failure to rescue (FTR). FTR is the loss of life among hospitalized patients resulting from the inadequate recognition and treatment of potentially fatal complications, and can be used as an important indicator of hospital care quality (25). Despite the assessment of frailty in head and neck cancer patients is a strong predictor of postoperative risk, the use of mFI-11 to prevent FTR in non-surgical patients is still scarce. Further studies should investigate the association between frailty and clinical complications during and after radiation and chemotherapy in cancer patients.

The mFI-11 has been validated in various surgical fields. In degenerative spine disease (26) and orthopedic surgeries (10), it has been found an association between frailty and major complications, prolonged length of stay and discharge, reoperation, and 30-day mortality. Similar results were found after colorectal cancer surgery (27), radical cystectomy in patients with bladder cancer (28), and after tumor resection in older gastrointestinal cancer patients (29). Surgery is a significantly stressful event that may induce a deteriorated physical status and depressed mood in the aged patient. Consequently, there is emerging evidence that the frailty index can identify patients at greatest risk for severe complications and mortality after oncologic surgery.

Although there are other approaches used to predict postoperative complications after head and neck surgery including the American Society of Anesthesiologists (ASA) classification and the Johns Hopkins Adjusted Clinical Groups (ACG) system, which are based on the evaluation of physical health and Fried physical phenotype model of frailty, respectively, the mFI-11 seems to be a more practical predictive tool because variables are easily assessed in the clinical setting and require less time in assessing frailty (8,10,19). Screening for frailty is important for good healthcare practice including patients having head and neck neoplasia. Recent consensus has recommended the assessment of frailty integrated into general clinical practice and not just limited to the application of instruments for screening the elderly population (30). Although mFI-11 is already applied with good results, improvements in this tool may be useful in the screening of frailty in many fields of medical and healthcare services.

The mFI-11 score can be applied using medical record databases (8) and can be calculated prospectively or retrospectively (31). In addition, the mFI-11 is especially useful in oncological head and neck patients because its variables have an impact on functional status (32). The dose-response analysis allows inferences regarding the association between different levels of exposure and the outcome of interest, and it has been recognized as one of the most important components of risk assessment. Investigating how the outcome risk varies throughout the exposure range can provide insights on the causal mechanism.

Recently, a categorical meta-analysis showed an association between frailty phenotype and postoperative complications among patients aged 60 years and over submitted to cardiac, gastrointestinal, and orthopedic surgeries (33). However, the frailty phenotype is limited to physical conditions and does not consider symptoms, signs, diseases, and disabilities as deficits. The mFI-11 recognizes that frailty is multi-factorial and dynamic. The results of this study suggest a linear dose-dependent effect between the mFI-11 score and major complications following head and neck oncologic surgery. Despite the data were fitted to linear and quadratic dose-response models, the linear function model was the best adjusted. We found a stepwise increase in RR of CD-IV and 30-day mortality for each additional point (comorbidity) on the mFI-11. Our results add further support to the understanding that cumulative effects of existing comorbidities may predict patients at high risk for major complications after head and neck oncologic surgery. The heterogeneity of patients with cancer and the multidimensional effects of both malignancy and surgery underscore the importance of incorporating more comprehensive preoperative assessments into cancer surgery (4).

This study has some major limitations. We included a heterogeneous group of tumors arising from the head and neck region, patients undergoing concomitant chemotherapy and radiotherapy, and a variety of surgical approaches. In addition, cancer surgeries may require more surgery time, which may be associated with an increased risk of complications. These characteristics may have a potential role in the between-study heterogeneity despite the choice of the random-effects model. Clinical or methodological diversity may be important sources of heterogeneity. In this sense, mFI-11 can be improved to capture these differences in head and neck cancer patients to mitigate heterogeneity and confer greater predictive value.

Although studies included in this systematic review had a low risk of bias, we recognize the potential for missing data in the medical records, the lack of comparisons with a physical frailty phenotype due to the limitations of the dataset, and the lack of long-term register-based follow-up. Moreover, further studies are needed to compare mFI-11 with other measures of comorbidities.

## Conclusions

Higher frailty scores are associated with a significant increase in ICU-level complications and 30-day mortality after head and neck oncologic surgery.
